# Ambient Air Pollution and Increasing Depressive Symptoms in Older Women: The Mediating Role of The Prefrontal Cortex

**DOI:** 10.1093/geroni/igab046.1318

**Published:** 2021-12-17

**Authors:** Andrew Petkus, Xinhui Wang, Diana Younan, Daniel Beavers, Mark Espeland, Joshua Millstein, Margaret Gatz, Jiu-Chiuan Chen

**Affiliations:** 1 University of Southern California, LOS ANGELES, California, United States; 2 University of Southern California, Los Angeles, California, United States; 3 Wake Forest School of Medicine, Winston-Salem, North Carolina, United States

## Abstract

Exposure to air pollution may accelerate brain aging and increase risk of late-life depressive symptoms (DS). Brain structures underlying these associations are unknown. Longitudinal data from 829 community-dwelling women without dementia (baseline age 81.6 ± 3.6 years old) who participated in both the Women’s Health Initiative Memory Study Magnetic Resonance Imaging study (WHIMS-MRI; 2005-06) and the WHIMS-Epidemiology of Cognitive Health Outcomes (2008-16) were analyzed to examine whether volumetric measures of brain structures mediated associations between long-term exposure to ambient air pollutants and annual increases in DS (as measured by annually assessed 15-item Geriatric Depression Scale). Annual PM2.5 (fine particulate matter of aerodynamic diameter <2.5) and NO2 were estimated at the participants’ residence using regionalized universal kriging models and aggregated to the 3-year average prior to the WHIMS-MRI. Structural equation models were constructed to estimate associations between exposure, structural brain variables, and trajectories of DS (standardized on baseline mean and SD). Living in locations with higher NO2 (standardized β = 0.023; 95% Confidence Interval (CI) = 0.004, 0.042) or PM2.5 (standardized β = 0.021; 95% CI = 0.004, 0.038) was associated with larger annual increases in DS (~60% larger annual increase in DS). Higher NO2, but not PM2.5, was associated with smaller prefrontal cortical volumes (standardized β = -0.431; 99% CI = -0.518; -0.344). Prefrontal cortical volume explained 30.4% of the total association between annual DS increase and NO2. These findings underscore the importance of the prefrontal cortex in associations between NO2 exposure and increasing DS in later-life.

